# A novel ultra-sensitive method for the detection of *FGFR3* mutations in urine of bladder cancer patients – Design of the Urodiag® PCR kit for surveillance of patients with non-muscle-invasive bladder cancer (NMIBC)

**DOI:** 10.1186/s12881-020-01050-w

**Published:** 2020-05-24

**Authors:** Jean-Pierre Roperch, Claude Hennion

**Affiliations:** OncoDiag, 9 rue de Pacy, 27930 Miserey, France

**Keywords:** MASO-PCR, NMIBC, Urodiag® PCR kit, Urine-based laboratory test, Surveillance, Mutation and methylation markers

## Abstract

**Background:**

We have recently developed a highly accurate urine-based test, named Urodiag®, associating *FGFR3* mutation and DNA methylation assays for recurrence surveillance in patients with low-, intermediate-, and high-risk NMIBC. Previously, the detection of four *FGFR3* mutations (G372C, R248C, S249C and Y375C) required amplification steps and PCR products were analyzed by capillary electrophoresis (Allele Specific-PCR, AS-PCR), which was expensive and time-consuming. Here, we present the development a novel ultra-sensitive multiplex PCR assay as called “Mutated Allele Specific Oligonucleotide-PCR (MASO-PCR)”, generating a cost-effective, simple, fast and clinically applicable assay for the detection of *FGFR3* mutations in voided urine.

**Methods:**

Comparative clinical performances of MASO-PCR and AS-PCR technologies were performed from 263 urine DNA samples (87 *FGFR3* mutated and 176 *FGFR3* wild-type). In the development of Urodiag® PCR Kit, we studied the stability and reproducibility of each all-in-one PCR master mix (single reaction mixture including all the necessary PCR components) for MASO-PCR and QM-MSPCR (Quantitative Multiplex Methylation-Specific PCR to co-amplify *SEPTIN9*, *HS3ST2* and *SLIT2* methylated genes) assays.

**Results:**

Complete concordance (100%) was observed between the MASO-PCR and AS-PCR results. Each PCR master mix displayed excellent reproducibility and stability after 12 months of storage at − 20 °C, with intra-assay standard deviations lower than 0.3 Ct and coefficient of variations (CV) lower than 1%. The limit of detection (LoD) of MASO-PCR was 5% mutant detection in a 95% of wild-type background. The limit of quantification (LoQ) of QM-MSPCR was 10 pg of bisulfite-converted DNA.

**Conclusions:**

We developed and clinically validated the MASO-PCR assay, generating cost-effective, simple, fast and clinically applicable assay for the detection of *FGFR3* mutations in urine. We also designed the Urodiag® PCR Kit, which includes the MASO-PCR and QM-MSPCR assays. Adapted to routine clinical laboratory (simplicity, accuracy), the kit will be a great help to urologists for recurrence surveillance in patients at low-, intermediate- and high-risk NMIBC. Reducing the number of unnecessary cystoscopies, it will have extremely beneficial effects for patients (painless) and for the healthcare systems (low cost).

## Background

Nowadays, the Bladder cancer (BCa) remains a serious public health issue. In 2018, the global incidence of BCa was estimated at around 550,000 new cases, ranking the disease as the 7th among cancers [1]. In the primary BCa diagnosis, the majority (> 70%) are non-invasive bladder cancers (NMIBC), including stages Ta, T1 and Tis (carcinoma in situ) and nearly 30% with muscle invasive bladder cancer (MIBC) (stages T2 to T4) [2]. The primary treatment of patients with NMIBC is transurethral resection of bladder tumor. Despite the treatment, more than 70% of them will develop a recurrence in 2 years (90% in 15 years) and therefore be followed by periodic cystoscopy and urinary cytology [2]. BCa is the most expensive of all cancers [3]. Cystoscopy is an uncomfortable invasive exam and cytology presents a very low sensitivity to detect NMIBC at low risk [4]. In this context, the development of reliable and affordable tools to detect recurrence is a challenge. Genetic and epigenetic alterations in DNA have been reported in the development and progression of bladder cancer [5]. In the mutational path, the fibroblast growth factor receptor 3 gene (*FGFR3*) appears to be the most frequently mutated gene in BCa. A dozen *FGFR3* mutations have been found in this disease [6, 7], but four of them (G372C, R248C, S249C, and Y375C) account for > 95% cases [8]. These four mutations were found in the urine and then proposed as a molecular tool for the diagnosis and monitoring of patients with NMIBC at low risk [9–11]. Likewise, epigenetic modifications, such as DNA hypermethylation, have been shown to play a key role in BCa [12–16]. Like Serizawa’s work [17], we have also shown that detection of *FGFR3* mutations combined with DNA methylation analysis could be is an excellent strategy to develop an accurate urine-based test in the surveillance of patients treated for NMIBC [18]. Here, we developed and clinically validated the MASO-PCR assay for the detection of *FGFR3* mutations in urine. We also presented the design of the Urodiag^®^ PCR Kit, a new urine-based lab test to monitor NMIBC patients with low-, intermediate and high-risk.

## Methods

### Urine collection and capture of exfoliated bladder cells with a membrane filter

Urine samples (100 ml) were collected (*n* = 26) from the first miction in the morning into a clean sterile container. Urine samples were pooled and stored at 4 °C for up to 72 h prior analysis. Each healthy donor gave consent before study participation. One hundred milliliter of each pooled urine sample were filtered through a single-use syringe-filter (Filter), presenting a nylon disc filter of 11 μm porosity and 25 mm diameter (Merck-Millipore). The Filter was rinsed with 5 ml of 1X PBS (pH 7.4).

### Urine DNA extraction

Urine DNA isolation has been carried out directly from bladder cells captured on Filter using the QIAamp DNA Blood Mini Kit (Qiagen). If the Filter has been stored at − 20 °C, it has to be left 5–10 min at RT be before DNA extraction. The lysis buffer (220 μl 1X PBS, 22 μl Proteinase K and 220 μl AL buffer) were added on the filter and then passed 3 times through a syringe equipped with a 21 gauge needle (Terumo) to shear genomic DNA. The lysate was incubated at 56 °C for 15 min into a 2 ml tube. Subsequent processing was done according to the DNA purification protocol. All centrifugation steps were carried out in a benchtop microcentrifuge (14,000 RPM) at RT. DNA was eluted from the column into a clean 1.5 ml tube by adding 50 μl of AE buffer to the column. DNA concentration was determined with Qubit 4 fluorometer (Invitrogen) and the highly sensitive Qubit quantification assay. All genomic DNA samples were diluted or concentrated to obtain a final concentration of 1.25 ng/μl. All samples were examined for DNA (5 ng) integrity via PCR amplification of the *GLOBIN* gene.

### Reproducibility and stability study

We studied the reproducibility of the urine filtration (17 urine samples belonging to Pool 1 to 4). We also studied the stability of filters, recovered after filtration (9 filters belonging to Pool 5 to 7), according to temperature and storage time. This study requires the following steps: DNA extraction from filter, DNA quantification and PCR amplification of the *GLOBIN* gene.

### Bisulfite DNA modification

Thirty nanograms of universal methylated human DNA standard (Zymo Research) were modified by the EZ DNA Methylation kit (Zymo Research) according to the manufacturer’s instructions. All the centrifugation steps were carried out in a benchtop microcentrifuge (14,000 rpm) at RT. The DNA modification was performed by incubation steps, at 37 °C for 15 min then at 50 °C for 15 h30 (overnight), in a thermal cycler. Bisulfite DNA conversion was eluted from the column tube by adding 10 μl of M-Elution buffer into a clean 1.5 ml.

### Multiplex real-time PCR

All PCR reactions were performed on the real-time PCR instrument StepOnePlus™ (Thermo Fischer Scientific). The PCR cycling parameters were: initial denaturation at 95 °C for 5 min followed by 40 cycles of 15 s at 95 °C, 45 s at 60 °C. The fluorescence data was acquired at the end of each cycle.

#### *FGFR3* mutation analysis using mutated allele specific oligonucleotide-PCR (MASO-PCR)

**Fig. 1:**
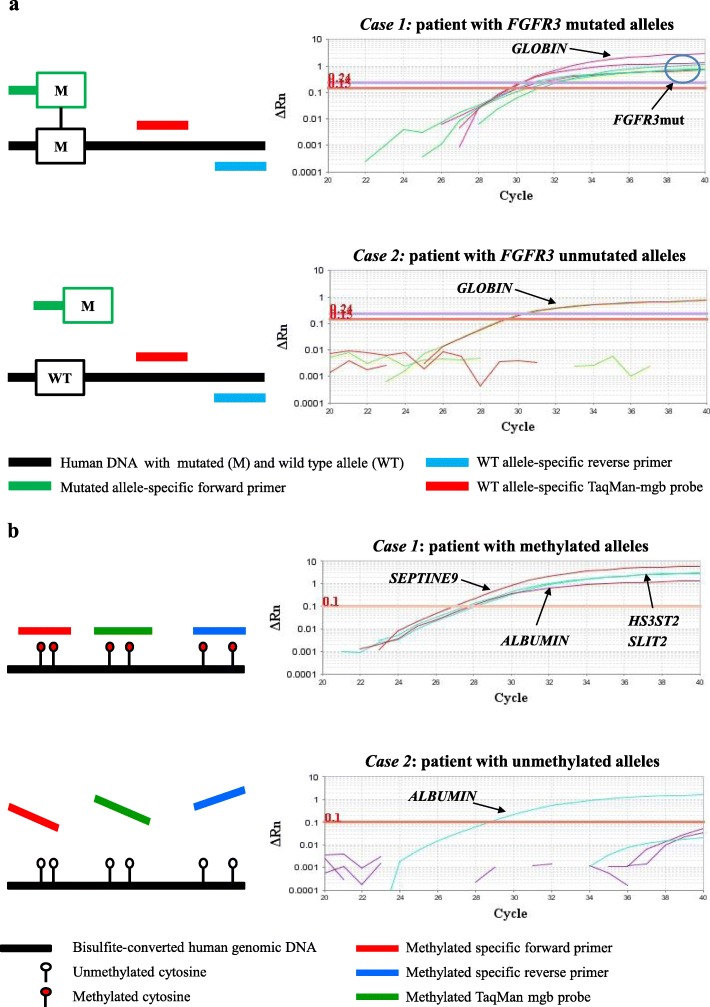
**a**–**b** Design of Mutation and Methylation PCR assays. The diagram 2a illustrates the Mutation assay with the position of the primers and fluorescent probes used for detection of human *FGFR3* mutations (G372C, R248C, S249C, and Y375C) by MASO-PCR. The diagram 2b illustrates the Methylation assay with the position of the primers and fluorescent probes used in quantifying methylation degree of *HS3ST2*, *SEPTIN9*, and *SLIT2* genes by QM-MSPCR. In both assays, the amplification curves are shown as examples, with Cts values above the established threshold as positive (*Case 1*) and below the threshold as negative (*Case 2*)

The MASO-PCR technology (Fig. 1a) was performed to simultaneously detect four mutations of the *FGFR3* gene (*FGFR3*mut) with 6Fam-S249C and Vic-Y375C (MASO-PCR1) and 6Fam-R248C and Vic-G372C (MASO-PCR2). PCR was conducted with 4 μl (5 ng) of DNA template and 16 μl 1X Quantifast Multiplex PCR (Qiagen), 500 nM of primers (Eurogentec) and 200 nM of TaqMan-mgb probes (Thermo Fischer Scientific). The DNA integrity has been checked by amplification of the Ned-*GLOBIN* gene. All primers and probes are listed in Table 1a

**Table 1:**
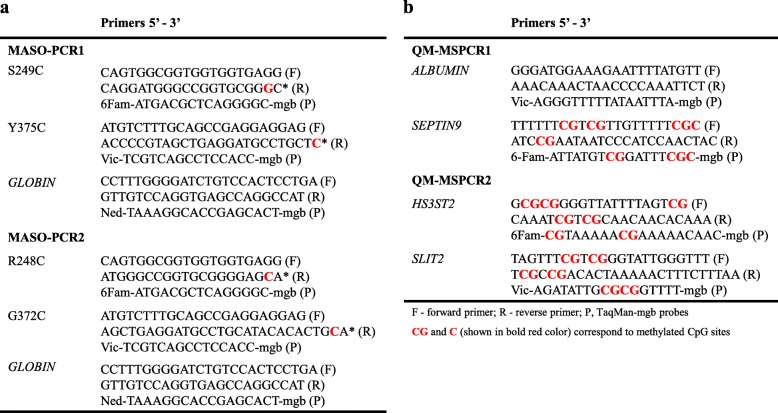
Primers sequences for MASO-PCR and QM-MSPCR

#### Methylation analysis using quantitative multiplex methylation specific-PCR (QM-MSPCR)

We performed two QM-MSPCR (Fig. 1b) for co-amplification of 6Fam-*SEPTIN9* with Vic-*ALBUMIN* (QM-MSPCR1) and 6Fam-*HS3ST2* with Vic-*SLIT2* (QM-MSPCR2). All reactions were performed with 4 μl of bisulfite-converted positive control DNA (100% methylated) and 16 μl of PCR mix containing 1x KAPA PROBE FAST qPCR Master Mix (KAPA Biosystems), 400 nM primers (Eurogentec) and 250 nM TaqMan-mgb probes (Thermo Fischer Scientific). *ALBUMIN* sequence has been designed without CpG site and used for normalizing the DNA amounts. All primers and probes are presented in Table 1b.

### Detection of FGFR3 mutations using MASO-PCR in patients with NMIBC

We selected 263 urine DNA samples, including 176 *FGFR3* wild-type (wt) and 87 *FGFR3*-mutated (mut) previously validated by AS-PCR, from NMIBC patients (AUVES cohort, project reference RECF0998-PHRC 2003) [11]. For initial diagnosis the distribution of patients (*n* = 57) among low (L)/intermediate (I) and, high (H)-risk NMIBC was 51%/23 and 26%. For follow-up the distribution of patients (*n* = 30) among L/I and H-risk NMIBC was 56%/17 and 27%. The distribution of mutations was: For initial diagnosis (*n* = 107): *FGFR3*mut (*n* = 57): S249C (*n* = 31), Y375C (*n* = 14), R248C (*n* = 7), G372C (*n* = 3) and R248C/S249C (*n* = 2), and *FGFR3*wt (*n* = 50). For follow-up (*n* = 156): *FGFR3*mut (*n* = 30): S249C (*n* = 20), Y375C (*n* = 4), R248C (*n* = 3), G372C (*n* = 2) and R248C/S249C (*n* = 1), and *FGFR3*wt (*n* = 126).

### MASO-PCR: *FGFR3* positive control, primer specificity, and determination of limit of detection (LoD)

#### Construction of the control plasmids containing *FGFR3* mutations

Positive control plasmids were designed to incorporate the *FGFR3* mutations into pMA-T vector (GeneArt, ThermoFisher Scientific). Each positive control plasmid was confirmed by sequencing before use.

- *FGFR3*mut plasmid n°1 (2571 bp): pMAT vector (2374 bp) + S249C and Y375C mutations (197 bp) (Additional file 1: Figure S1a)
- *FGFR3*mut plasmid n°2 (2560 bp): pMA-T vector (2374 bp) + R248C and G372C mutations (186 bp) (Additional file 1: Figure S1b)

#### Primer pair specificity

We used the same *FGFR3* primer pairs (Table 1a) to amplify *FGFR3* mutations with the Fast SYBR Green PCR master mix (SG-PCR, ThermoFisher Scientific). PCR reactions (20 μl) were performed, in duplicate onto two separated runs, with a 1X SG (10 μl), 200 nM of primers and *FGFR3*mut plasmid (4 μl). The thermal cycling conditions included an initial denaturation at 95 °C for 3 min followed by 40 cycles: 95 °C for 3 s and 60 °C for 20 s. The melting temperature (Tm) of each amplicon was calculated by the StepOnePlus software (Life Technologies) and also estimated by Howley’s formula: [67.5 + (0.41* %G-C) - (395/length of amplicon)].

#### LoD for analysis of *FGFR3* mutations

The diploid human genome comprises about 6.10^9^ base pairs (bp). Plasmids (2.50 ng/μl) were diluted at 2.10^6^ in the standard human DNA (*FGFR3*wt, 2.50 ng/μl), leading to a 1:1 ratio (*FGFR3*mut/*FGFR3*wt) and dilutions were used as *FGFR3* positive controls. To determine the LoD, a serial dilution series of the each *FGFR3* positive control was produced at 50, 10, 5, and 1% with *FGFR*3wt (1.25 ng/μl). 5 ng of each dilution were amplified by MASO-PCR with a predefined positive threshold (ΔRn) at 0.15 for *GLOBIN*, S249C, Y375C, G372C and 0.24 for R248C. All dilutions were amplified and then analyzed in duplicate on the same plate to PCR in two independent runs.

### QM-MSP: positive control and determination of the limit of quantification (LoQ)

The LoQ of each gene was determined for QM-MSPCR1 and QM-MSPCR2 by performing a dilution range with 10, 1, 0.1 and 0.01 ng of bisulfite-converted positive control DNA (100% methylated). The limit of DNA quantity and amplification efficiency (E) were analysed using a threshold value (ΔRn) of 0.10. Each dilution was done in duplicate on the same PCR plate in two independent runs.

### Stability and reproducibility study of all-in-one PCR master mixes

The all-in-one PCR master mixes were prepared in a single reaction mixture including all PCR components for mutation and methylation assays. We studied the stability of each all-in-one PCR master mix by MASO-PCR1,2 and QM-MSPCR1,2 from aliquots that were run in triplicate and stored at − 20 °C for 0, 1, 2, 3, 4, 6, 9 and 12 months.

## Results

### Reproducible and efficient DNA extraction from bladder cells captured on a membrane filter

**Fig. 2:**
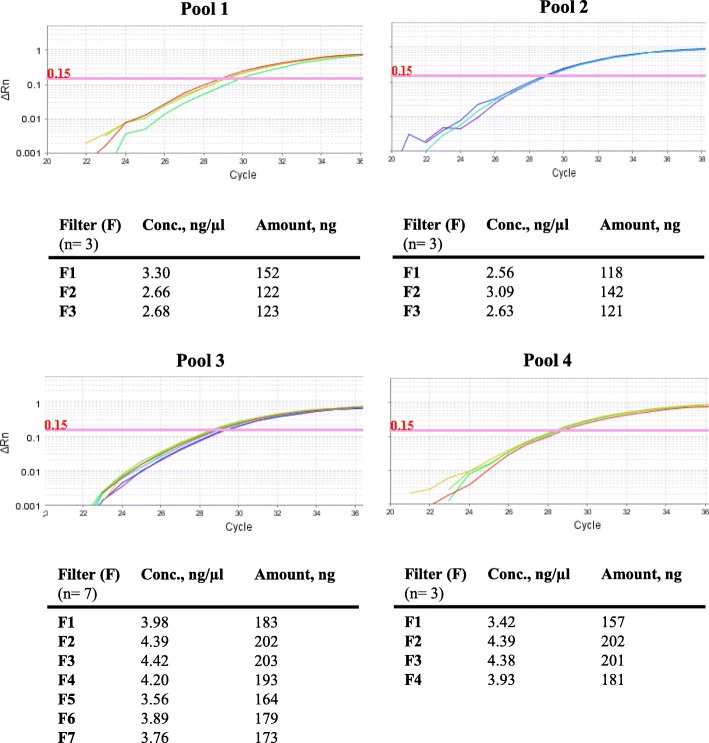
DNA integrity assessed by PCR amplification of *GLOBIN* gene. DNA concentrations were determined by fluorometry. The *GLOBIN* gene was amplified with an amount of urine DNA comprised between 10 and 18 ng (4 μl of DNA sample) from each Filter (F). Amplification curves are shown from Pool 1 to Pool 4, respectively

To assess if filtration allows capturing the fraction of bladder cells in the sample, DNA was isolated and amplified using real-time PCR. Urine filtration was reproducibly obtained for 17 pooled urine samples belonging to Pool 1 to 4. In Fig. 2, we have reported the amounts of DNA (mean ± standard deviation) recovered from each filter (F) with 132 ± 17 ng (Pool 1, *n* = 3), 128 ± 13 ng (Pool 2, n = 3), 187 ± 15 ng (Pool 3, *n* = 7) and 185 ± 21 ng (Pool 4, *n* = 4). The integrity of each extracted urinary DNA (10 ng) was confirmed by amplification of the *GLOBIN* gene.

**Fig. 3:**
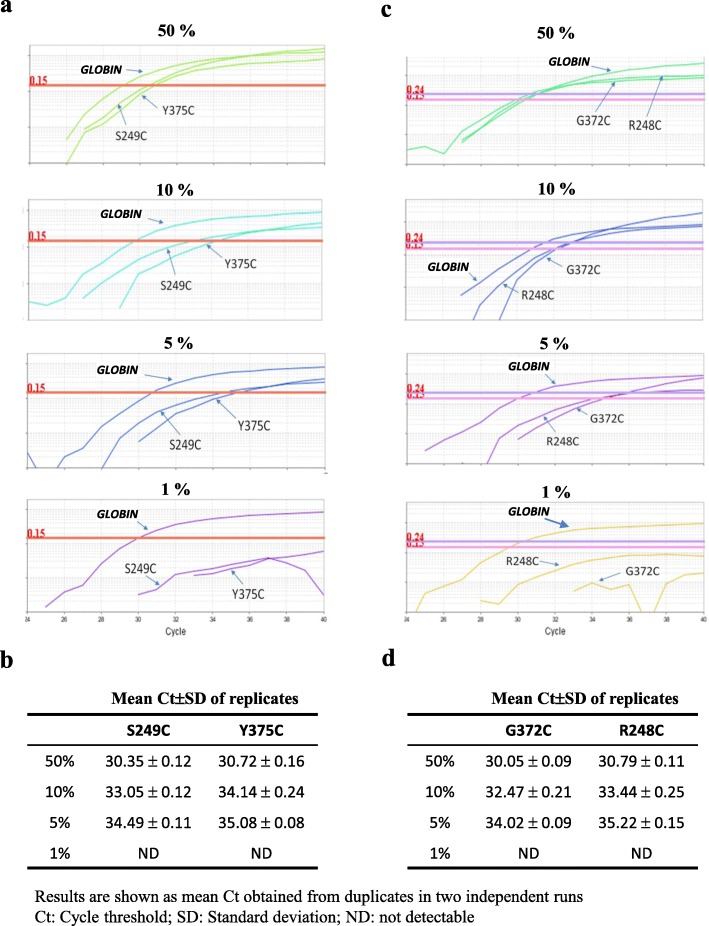
LoD for the Mutation assay. DNA from *FGFR3* mutant plasmids was diluted into the wild-type DNA (standard human DNA). The proportion of mutant DNA was 50, 10, 5, and 1%, respectively. The representative amplification curves (**a**, **c**) and mean Ct values (**b**, **d**) are shown in the detection of the *FGFR3* S249C/Y375C (a, b) and R248C/G372C (**c**, **d**) mutations by MASO-PCR

**Fig. 4:**
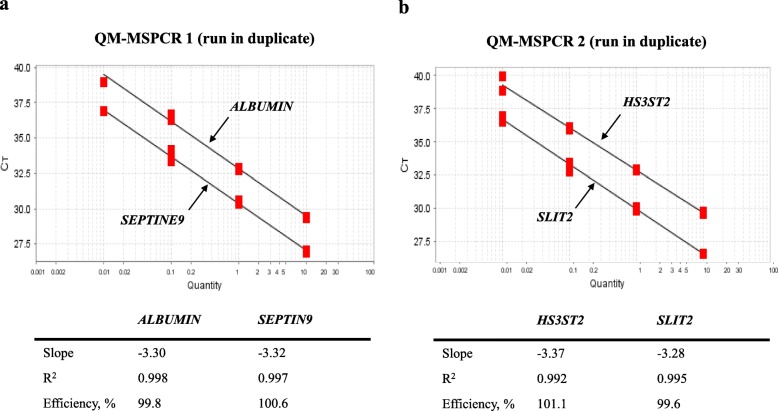
LoQ for the Methylation assay. The limit of quantification (LoQ) for the QM-MSPCR1 (**a**) and QM-MSPCR2 (**b**) was determined by carrying out a series of dilutions with bisulfite converted DNA quantities of 10, 1, 0.1 and 0.01 ng

### Effects of filter storage conditions

Concentration and recovery rate of the genomic DNA (Pools 5 to 7) according to filter storage conditions were summarized in Table 2. Filters of groups B (5 days at room temperature, RT) and C (5 days at − 20 °C) were compared to filters belonging to group A (0 day of storage). No significant differences were found among A and C, but there is a significant difference between A and B. Indeed, the DNA yields of groups A, B and C were 100%, 54 ± 13% (mean ± standard deviation) and 112 ± 23%, respectively. We have successfully verified the integrity of each isolated DNA by amplifying a segment of the *GLOBIN* gene. The amount of DNA obtained under all these conditions was greater than 40 ng, corresponding to the amount required to perform the test. In this study, we showed that the filter could be stored for 5 days at RT and, if a long-term storage is required until DNA extraction, optimal conditions are obtained at − 20 °C.

**Table 2:**
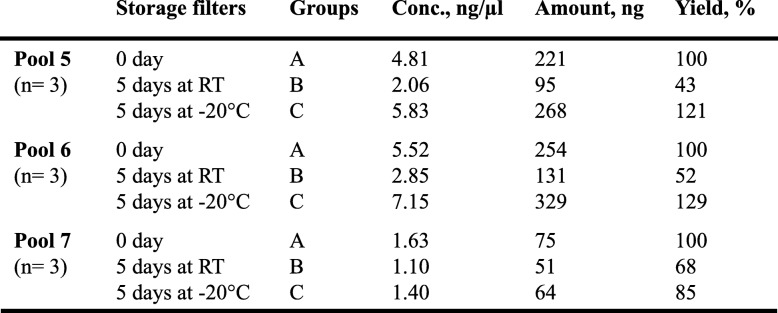
Relationship between filter storage conditions, concentration and amount of urine DNA

### Validation of primer pairs for the detection of *FGFR3* mutations by MASO-PCR

The melting curves for G372C, R248C, S249C, and Y375C mutations by SG-PCR are represented in additional file (Figure S2). The temperature of melting (Tm) for each amplicon was 83.81 ± 0.01 °C for G372C, 86.80 ± 0.02 °C for R248C, 87.54 ± 0.01 °C for S249C and 84.85 ± 0.02 °C for Y375C. By applying, the Howley’s formula, we obtained equivalent Tm as compared with those given by the melting curves, with 83.87 °C for G372C (63.7% G-C, 72 bases), 86.85 °C for R248C (71.8% G-C, 78 bases), 87.57 °C for S249C (73.2% G-C, 82 bases) and 84.77 °C for Y375C (65.5% G-C, 79 bases), respectively. These two methods allowed us to validate the specificity of each primer pair.

### Sensitive detection of *FGFR3* mutations by MASO-PCR

The limit of detection (LoD) of the Mutation assay with 5 ng was set at 5% of mutant sequences in a background of 95% normal DNA (Fig. 3a-b). This means that in the presence of a DNA sample containing less than 5% mutant (~ 15 copies), the MASO-PCR would be unable to detect the 4 mutations of the *FGFR3* gene (G372C, R248C, S249C, and Y375C). The positive reactions (amplification curves) were carried out in duplicate onto two separate runs with a very good reproducibility. Cts were obtained with cut-off values (ΔRn) of 0.15 for *GLOBIN*, G372C, S249C, Y375C and 0.24 for R248C (Fig. 3c-d).

### MASO-PCR accurately predicts recurrence of patients with NMIBC

By applying these threshold values, we clinically validated the MASO-PCR technology from 263 urine DNA samples (87 *FGFR3* mutated and 176 *FGFR3* wild-type). A complete concordance (100%) was observed between the MASO-PCR as compared with AS-PCR results. Sensitivity was defined as the ability of the MASO-PCR assay to detect *FGFR3* mutations (+) and specificity as the ability of assay to identify the absence of *FGFR3* mutations (−) in primary NMIBC tumor (diagnosis) as well as recurrence (follow-up). We successfully demonstrated the capacity of the MASO-PCR assay for detecting at least 15 copies of *FGFR3* mutant alleles in 5 ng of wild type DNA with a sensitivity and specificity of 100% in urine of patients with low-, intermediate- and high-risk. All data are shown in Table 3.

**Table 3:**
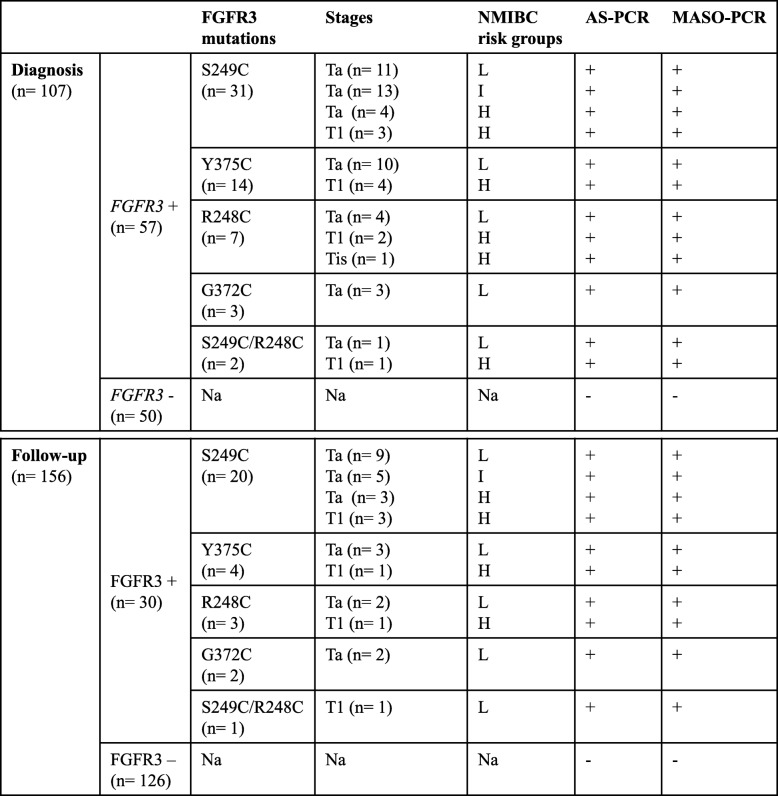
Ultra-sensitive MASO-PCR method for surveillance of NMIBC patients

### Sensitive quantification of DNA methylation by QM-MSPCR

QM-MSPCR was used to amplify *ALBUMIN*/*SEPTIN9* (QM-MSPCR1) and *HS3ST2*/*SLIT2* (QM-MSPCR2) duplexes with titration of bisulfite-converted positive control DNA (100% methylated) at various concentrations (10, 1, 0.1, 0.01 ng/well). At each dilution, the cycle threshold (Ct) was determined with bisulfite-converted positive control DNA (100% methylated). The Cts were analysed by using threshold value (ΔRn) of 0.10. Both calibration curves gave a slope of about − 3.32, which corresponds to PCR efficiency (E) close to 100%. More precisely, the slope values were − 3.31, − 3.34, − 3.29, and − 3.30 for *ALBUMIN* (E = 100.5%), *SEPTIN9* (E = 99.2%), *HS3ST2* (E = 101.4%). and *SLIT2* (E = 100.8%), respectively. These results reflect very high amplification efficiency. We determined that the limit of quantification (LoQ) of each target gene could be detected with 10 pg of DNA. In addition, the high value of the correlation coefficient (greater than 0.99) indicates that an almost perfect linearity is obtained over the entire range. Data are represented in Fig. 4a for QM-MSPCR1 and Fig. 4b for QM-MSPCR2.

### High stability and reproducibility of “all-in-one” PCR master mixes

The all-in-one PCR master mixes allow using solutions containing all the necessary reagents for PCR amplification of DNA. In Table 3, Ct values of each target gene are indicated in function of storage time of the MASO-PCR and QM-MSPCR solutions. We have successfully verified the reproducibility and stability of each all-in-one solution after 12 months of storage at − 20 °C, showing intra-assay standard deviations lower than 0.3 Ct and coefficient of variations (CV) lower than 1% (Table 4).

**Table 4:**
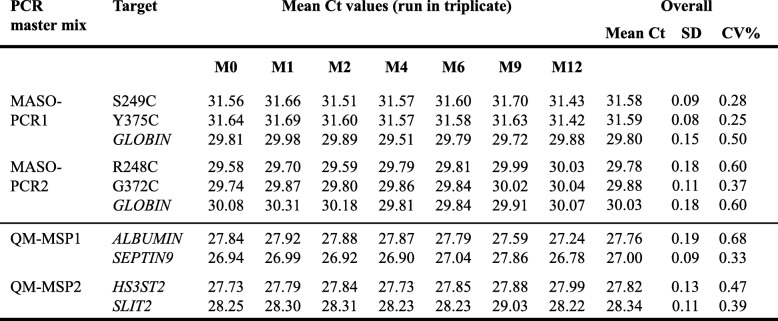
High performance of PCR Master mix all-in-one

### Design of Urodiag^®^ PCR kit

The Urodiag^®^ PCR Kit is an in vitro diagnostic test intended for the qualitative detection of *FGFR3* somatic mutations (G372C, R248C, S249C, Y375C) and the quantification of three DNA methylation markers (*HS3ST2*, *SEPTIN9*, *SLIT2*) by stable multiplex PCR in urine of NMIBC patients. The PCR kit is composed of 8 tubes (4 for the Mutation assay, 3 for the Methylation assay and 1 tube with sterile water) (Table 5). Each tube contains all the components (PCR mastermix, primers and probes) necessary to carry out Mutation and Methylation assays.

**Table 5:**
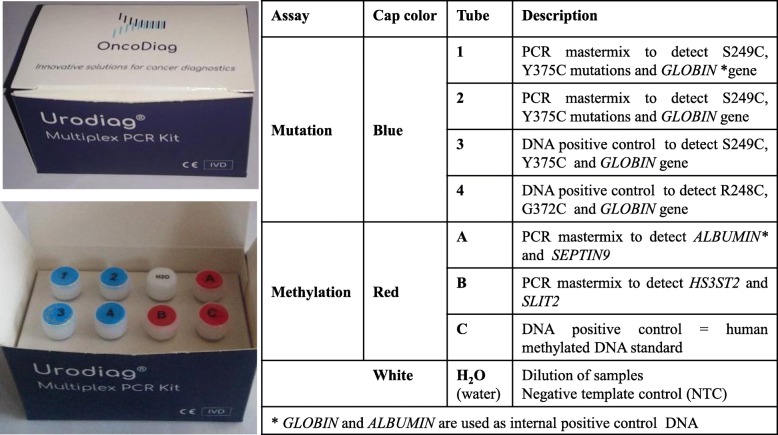
Components of the Urodiag® PCR Kit

## Discussion

Due to the high recurrence rate of bladder cancer, NMIBC tumors require a active surveillance: periodic cystoscopy with urine cytology remains the reference examination, making it the most expensive of all cancers [19]. Cystoscopy is an uncomfortable invasive exam and cytology presents a good sensitivity for detecting high-risk NMIBC but a very poor sensitivity for low-risk [20]. Urine is an ideal biological source for recovering bladder cells and mainly exfoliated tumor cells [21]. Previous work has shown that filtration of urine samples, using a syringe filter with a suitable pore size, increases the diagnostic accuracy of BCa while removing contaminant (e.g. blood cells) [21, 22]. In order to optimize the accuracy of our test, the filtration of urine samples was carried out by a disposable syringe filter device. Currently, urine tests are available for primary diagnosis and follow-up of patients with NMIBC such as ADXBLADDER test ($52 per test), Bladder Epicheck test (not yet marketed), bladder tumor associated antigen (BTA, $40 per test), ImmunoCyt ($200 per test), nuclear matrix protein 22 (NMP22, $25 per test), UroVysion ($800 per test), Xpert BC Monitor ($165 per test) [23–30]. Due to their lack of specificity or sensitivity, these tests are not widely used in routine laboratory. Prior studies have reported that some mutations of *FGFR3* gene are mainly found in NMIBC (~ 60%) versus MIBC (~ 20%) [31, 32]. In NMIBC, the four most relevant mutations are found in exons 7 and 10 with S249C and R248C in exon 7, and Y375C and G372C in exon 10 [33, 34]. Zuiverloon and colleagues described that these four mutations can be detected in urine and used to develop a non-invasive test for the diagnosis and monitoring of patients with low-risk NMIBC [34]. Furthermore, it has been shown that high-risk tumors have generally more hypermethylated genes than low-risk tumors [35]. Consistently with all these observations, we were able to propose a panel of genetic (*FGFR3* mutations) and epigenetic (hypermethylation of the *HS3ST2*, *SEPTIN9* and *SLIT2*) urinary markers which, due to their strong complementarity, given a very high clinical accuracy for the monitoring in NMIBC Patients with a low, intermediate and high risk of recurrence [18]. In comparison with the tests above mentioned, our combined test gives the best clinical performances: sensitivity/specificity/NPV respectively equal to or greater than 95%/76%/99% [18]. During the present study, we designed the Urodiag^®^ kit so that it contains all the components of the PCR for its use in clinical routine. To increase the diagnostic accuracy of BCa, we showed the feasibility of enriching the exfoliated bladder cells with a unique syringe filter to replace traditional centrifugation. We showed that this device was able to isolate DNA with reproducibility, high purity and sufficient quantity for subsequent MASO-PCR and QM-MSPCR amplification. We have developed and clinically validated the MASO-PCR to detect four mutations of *FGFR3* gene (G372C, R248C, S249C and Y375C) with outstanding accuracy with 100% sensitivity/specificity, equivalent to the results that can be obtained using capillary electrophoresis for DNA analysis (AS-PCR). Consequently, the mutation and methylation assays can be carried out on the same real time quantitative PCR machine, facilitating the implementation of the Urodiag^®^ Kit in laboratories. To simplify the PCR workflow, we prepared the all in one master mixes, solutions containing all the necessary reagents for MASO-PCR and QM-MSPCR PCR, with two main advantages: reduction of pipetting errors and time saving.

## Conclusions

We showed that the Mutation assay (MASO-PCR) and Methylation assay (QM-MSPCR) could be simultaneously performed on the same real time quantitative PCR machine, facilitating the implementation of the Urodiag^®^ PCR Kit in laboratories. It has been designed as a urine-based laboratory test that provides a simple, fast, reliable and low-cost (~ $100 per test) method for diagnosis and individualized surveillance for patients with low-, intermediate- and high-risk NMIBC. Leading to a significantly reduction of repetitive cystoscopies, it presents major benefits for the quality of life of the patients during their follow-up, the work of the urologists and in terms of cost reduction for health care systems.

## Supplementary information

**Additional file 1: Figure S1.** Construction of mutated *FGFR3* plasmids

**Additional file 2: Figure S2.** Melting curve PCR analysis for the *FGFR3* mutations

## Data Availability

The datasets used and/or analysed during the current study are available from the corresponding author on reasonable request.

## Abbreviations

BCa: Bladder cancer
Ct: Cycle threshold
CV: Coefficient of variation
MASO-PCR: Mutated Allele Specific Oligonucleotide-PCR
LoD: Limit of detection
LoQ: Limit of quantification
NMIBC: Non-muscle invasive bladder cancer
NPV: Negative predictive value
PBS: Phosphate buffered saline
QM-MSPCR: Quantitative Multiplex-Methylation Specific PCR
RT: Room temperature
