# Supplementary material for: A novel ultra-sensitive method for the detection of FGFR3 mutations in urine of bladder cancer patients – Design of the Urodiag® PCR kit for surveillance of patients with non-muscle-invasive bladder cancer (NMIBC)
Source: BMC Med Genet. 2020 May 24;21:112. doi: 10.1186/s12881-020-01050-w (PMC7247276; doi:10.1186/s12881-020-01050-w)
Supplement: Supplementary file 1 — Additional file 1: Figure S1. Construction of mutated FGFR3 plasmids [file 12881_2020_1050_MOESM1_ESM.ppt]

## Slide 1
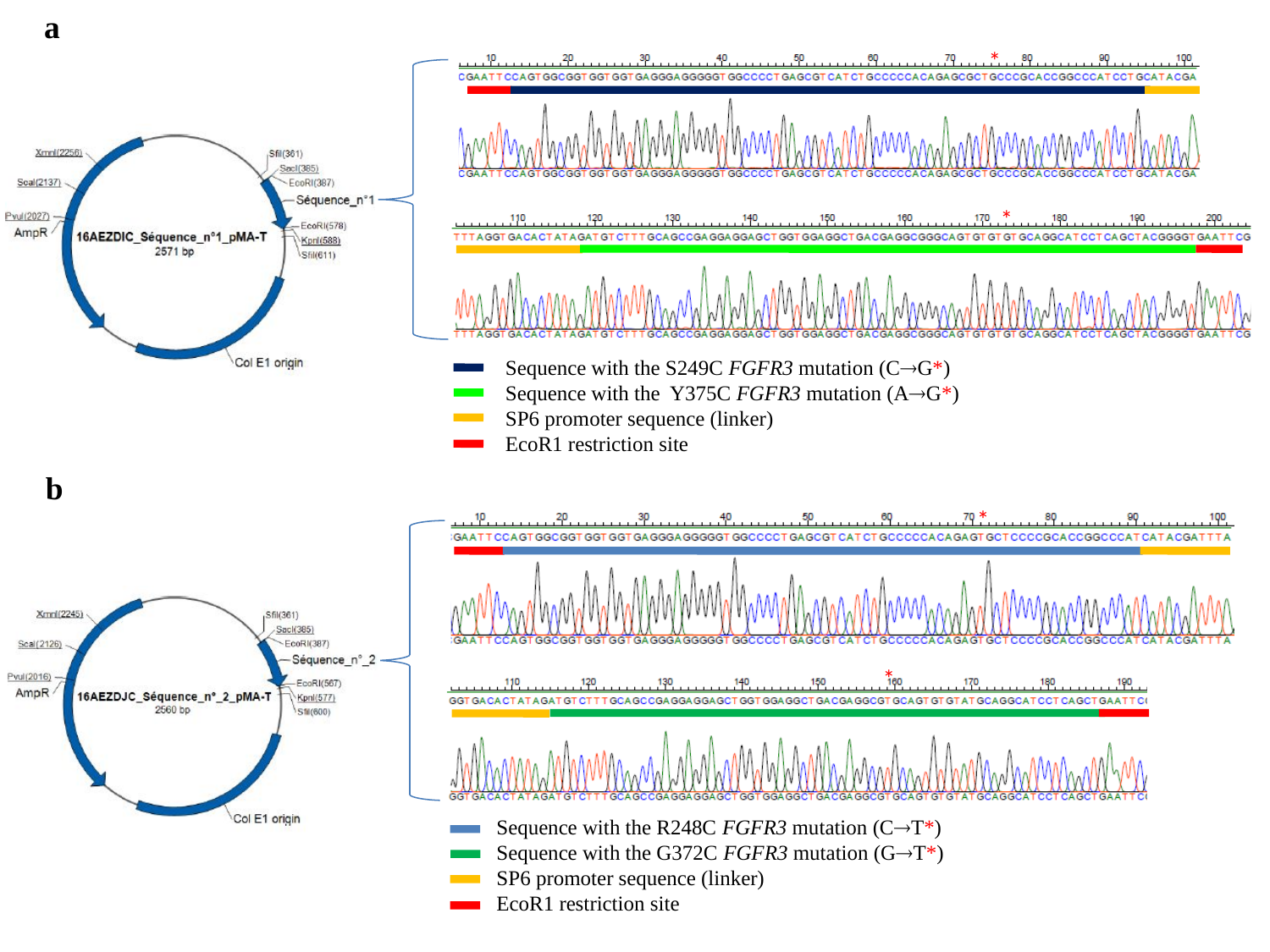

a
*
*
Sequence with the S249C FGFR3 mutation (CG*)
Sequence with the Y375C FGFR3 mutation (AG*)
SP6 promoter sequence (linker)
EcoR1 restriction site
b
*
*
Sequence with the R248C FGFR3 mutation (CT*)
Sequence with the G372C FGFR3 mutation (GT*)
SP6 promoter sequence (linker)
EcoR1 restriction site
