# Supplementary figures and images for: A novel ultra-sensitive method for the detection of FGFR3 mutations in urine of bladder cancer patients – Design of the Urodiag® PCR kit for surveillance of patients with non-muscle-invasive bladder cancer (NMIBC)
Source: BMC Med Genet. 2020 May 24;21:112. doi: 10.1186/s12881-020-01050-w (PMC7247276; doi:10.1186/s12881-020-01050-w)

## Slide 1
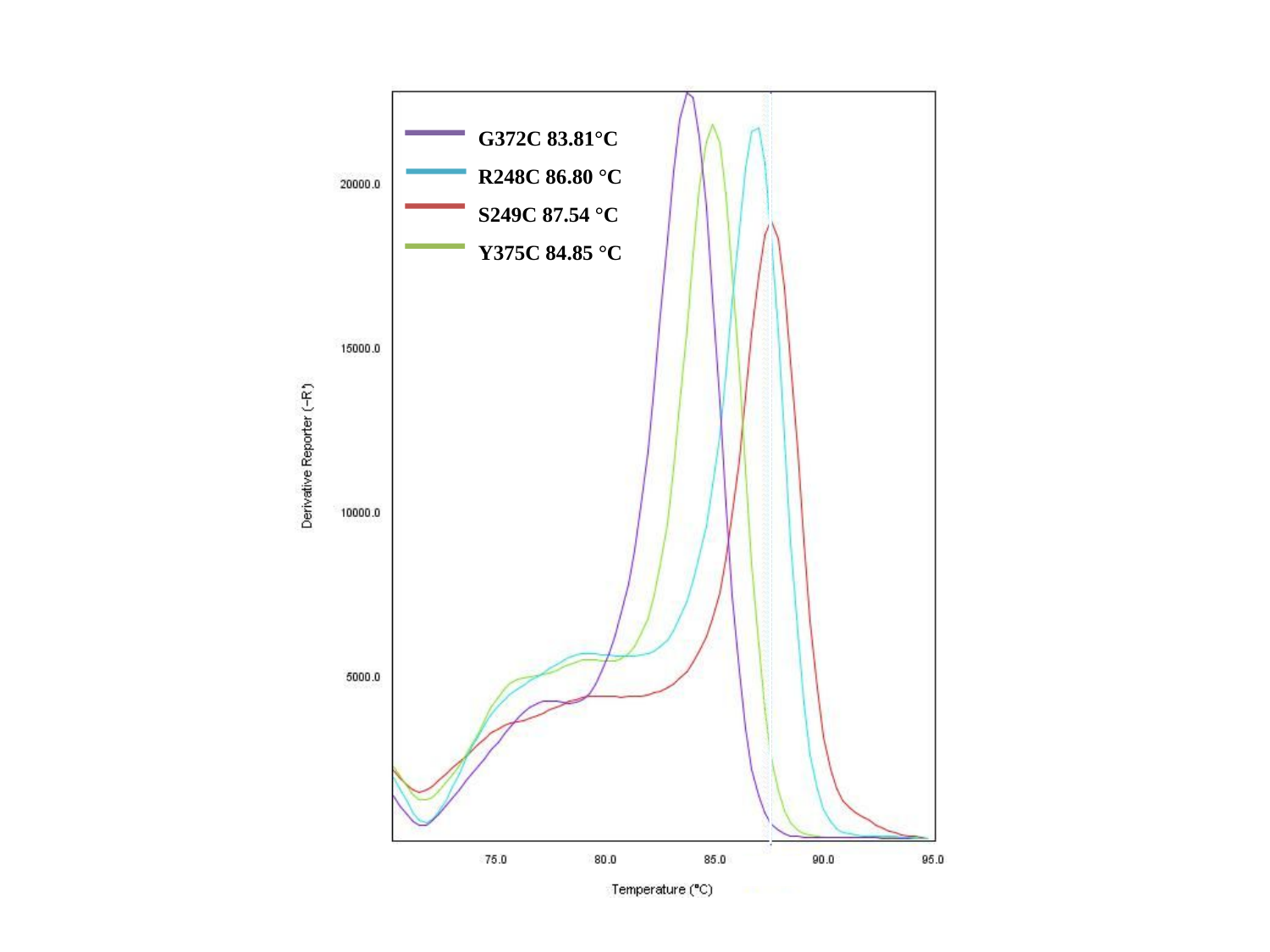

G372C 83.81°C
R248C 86.80 °C
S249C 87.54 °C
Y375C 84.85 °C

Supplement: Supplementary file 2 — Additional file 2: Figure S2. Melting curve PCR analysis for the FGFR3 mutations [file 12881_2020_1050_MOESM2_ESM.ppt]
